# Unveiling healthcare disparities in Somalia: the hidden struggles of nomadic communities

**DOI:** 10.1186/s12939-026-02846-7

**Published:** 2026-04-21

**Authors:** Hodan A. Duale, Abdulwahab M. Salad, Said Mohamoud, Abdirisaq M. Artan, Abdiweli A. Mohamed, Abdi Gele

**Affiliations:** 1 Somali Institute for Health Research, Mogadishu, Somalia; 2 Ministry of Health, Dhusamareb, Galmudug State Somalia; 3 Ministry of Health, Garowe, Puntland State Somalia; 4 Norwegian Institute of Public Health, Oslo, Norway; 5 Somali Institute for Health Research, Hargeisa, Somaliland; 6 Somali Institute for Health Research, Garowe, Somalia

**Keywords:** Pastoralists, Inequity, Somalia, Access, Healthcare

## Abstract

**Background:**

In Sub-Saharan Africa, there is substantial evidence indicating that health systems often overlook the healthcare needs of pastoralists and fail to adequately tailor services to their nomadic lifestyle. Although there are a few studies highlighting disparities in healthcare access across various communities in Somalia/Somaliland, there is limited knowledge about how pastoralists perceive and access health services. The aim of this study is to evaluate the inequity in healthcare access and explore challenges faced by pastoralists in accessing healthcare, with a focus on the Galmudug and Puntland states and Somaliland.

**Methods:**

The study employed a combination of quantitative and qualitative data to gather comprehensive insights into the healthcare access of pastoral communities. We conducted an analysis of the 2020 Somalia Demographic Health Survey (SDHS) to understand the healthcare utilization of pastoralists. Subsequently, we conducted a qualitative study using in-depth interviews (IDIs) involving 45 pastoralists. In analyzing the SDHS data, we employed descriptive methods such as frequency analysis, using SPSS software for data analysis. The results were presented in tables, which facilitated clear and concise visualization of the data. Regarding qualitative data analysis, we drew inspiration from the Tanahashi framework for coverage. The Tanahashi framework assesses the availability, accessibility, acceptability, and affordability of services.

**Results:**

The findings show that the majority of pastoralists have difficulties in securing funds for treatment (71.9%) and difficulties in accessing health facilities due to distance (74%). These figures are considerably higher compared to urban populations, with 58.5% and 51%, respectively, and rural populations, with 66% and 62%, respectively. Moreover, the qualitative study identified nine themes, which were organized under the four categories of the Tanahashi framework. Among the themes were eight supply-related themes such as struggling without services, the devastating impact of service shortfalls, transportation in emergency situations, distance to health facilities, mobile lifestyle, the financial strain of transport, being priced out of healthcare, and the sacrifice of livestock for health. Additionally, one theme related to demand was ‘culturally connected healthcare providers’.

**Conclusion:**

These findings underscore the urgency for comprehensive health system reforms in Somalia\Somaliland that fully incorporate pastoralist needs into health planning strategies, addressing the stark inequities in healthcare access experienced by pastoral communities.

**Clinical trial number:**

Not applicable.

**Supplementary Information:**

The online version contains supplementary material available at 10.1186/s12939-026-02846-7.

## Background

Nomadic pastoralist communities in Africa encounter significant health challenges, evidenced by their elevated rates of illness and mortality from preventable diseases [1]. These challenges have been a concern since the late 1970s, especially following the smallpox eradication efforts. Notably, outbreaks of smallpox were discovered among nomadic groups in Somalia, years after the disease was initially thought to have been eradicated. This highlighted the unique vulnerabilities and healthcare needs of nomadic populations, underscoring the importance of developing tailored health strategies to effectively address these challenges [1]. Today, various disease elimination and eradication programs, such as those targeting measles, tuberculosis and polio, continue to highlight the unique health dynamics associated with nomadic communities [2]. The movement of these communities between regions can contribute to the spread of diseases, positioning them both as potential reservoirs and vectors [3]. This mobility complicates the implementation of standard health interventions and requires tailored strategies to effectively address the health needs and challenges of nomadic pastoralist populations across Africa [3].

Somalia is experiencing decades of civil war that destroyed not only institutions but also governance standards and regulations. Given decades of clan conflict, Somalia adopted a clan power-sharing formula known as the 4.5 system as a means of political settlement and reconciliation among clans [4], . In the absence of other political structures, such as political parties or election results, this formula has increasingly become central to the distribution of power across various levels of government. Therefore, one of the significant obstacles in establishing an effective healthcare system in Somalia stems from the pervasive issue of political clannism, particularly concerning the appointment of key positions in the health sector [5]. Instead of appointing individuals based on competence and expertise, decisions are often influenced by the clan-based power-sharing system that the country has adopted to diffuse clan tensions [4]. The 4.5 system prioritizes political allegiance and clan affiliation over qualifications and experience, leading to the placement of individuals who may lack the necessary skills and knowledge to lead critical departments and programs effectively. This situation creates a cascading effect that impacts the overall functioning of the healthcare system by hindering strategic planning, policy development, and the implementation of effective health interventions.

Consequently, the healthcare system in Somalia is experiencing considerable challenges in developing and implementing tailored strategies that effectively address the diverse health needs of its population. A homogeneous approach to healthcare design, which fails to consider the unique characteristics of different groups, has led to significant gaps in healthcare delivery and exacerbated health disparities among communities [6]. Particularly affected are the nomadic pastoralists, whose specific healthcare challenges underscore the inadequacies within the nation’s overall health infrastructure [7]. Research has repeatedly demonstrated that marginalized communities such as pastoralists tend to experience lower survival rates and reduced utilization of healthcare services [7, 8]. Therefore, to attain equitable coverage, it is essential for healthcare systems to extend their reach to every community, particularly those that are marginalized [7, 9, 10].

Pastoralists are nomadic or semi-nomadic communities whose livelihoods primarily rely on livestock rearing. They travel from one location to another in search of water and suitable grazing areas for their animals. Although studies mention that their migratory culture have significantly changed last few decades due to the climate change that forced many pastoralists to settle in villages [7, 11], evidence indicates that even before turning to transhumance, their migration patterns were predictable, with an argument that health facilities can be made available for them if its designed in strategic locations that aligns with their seasonal migration [12]. A study in Somalia stressed that pastoral migration evolves in response to environmental variability, climate change-related droughts, and conflicts [13]. The frequent droughts in the country over the past decades have resulted in limited access to grazing land, leading to livestock deaths. Consequently, some pastoralists have been prompted to seek alternative livelihoods, potentially transitioning from a purely pastoralist lifestyle to a transhumant one [13]. Despite their mobility status, it has been found that the healthcare needs of pastoralists in Somalia are grossly neglected [7]. Generally, delivering healthcare to pastoralist communities in remote areas presents unique, complex logistical, structural, and cultural challenges. The core difficulties stem from the mobility of these populations, harsh environmental conditions, and limited infrastructure, which often result in these groups being among the least served in terms of public health [14]. Additionally, resource allocation issues, stemming from inadequate funding and insufficient staffing of facilities in pastoralist dominated areas, culminate in insufficient healthcare coverage for these communities [7]. This oversight leaves pastoral communities vulnerable, as their nomadic lifestyle requires flexible healthcare solutions that can adapt to their unique circumstances. To our knowledge, apart from older studies conducted prior to the 1990s, there is only a single study that has explored pastoralists’ access to healthcare in Somalia. However, that study was limited in scope, focusing solely on a village called Daresalam [7]. This study aims to evaluate the inequity in healthcare access and explore challenges faced by pastoralists in accessing healthcare using Tanahashi framework for health coverage [15]. It will contribute to the existing literature by offering an in-depth analysis of the healthcare access challenges faced by pastoralists and provides valuable insights to aid policymakers and stakeholders in developing targeted interventions and policies.

## Methodology

The study employed a multifaceted approach to gather comprehensive insights into healthcare provisions and needs within pastoral communities. We conducted analysis of the 2020 Somalia Demographic Health Survey (SDHS) to understand healthcare utilization of pastoralists. Afterwards, we conducted a qualitative study using in-depth interviews (IDIs) involving 45 pastoralist participants.

### Study setting

The study was carried out across three distinct regions: Puntland and Galmudug in northeastern and south-central Somalia respectively, and Somaliland. Puntland and Somaliland are recognized as peaceful regions and are noted for having the strongest health systems compared to states in southern Somalia. Conversely, though Galmudug is considered the most stable state in southern Somalia, it still experiences sporadic conflicts, primarily in districts controlled by non-state actors. We aim to investigate healthcare accessibility for pastoralist communities in regions that potentially offer more stable but diverse environments.

### Recruitment and data collection

In selecting participants, we adopted purposive sampling, specifically maximum variation sampling, which allowed us to capture a diverse range of individuals in terms of gender, age, settlement area, and their position within the community.

Data collection was carried out by a team of pre-trained research assistants across the three regions. Data collectors were trained in interview techniques, including how to establish rapport with participants, ask open-ended questions, and probe for deeper insights, as well as listening skills and ways to encourage participants to share their experiences and perspectives without leading or influencing their responses. In Puntland, the data was gathered by a male medical doctor. In Somaliland, the team comprised a male medical doctor along with two female medical students. In Galmudug, a male researcher was responsible for collecting the data. Using a semi-structured interview guide (appendix 1), we engaged participants with a series of open-ended questions designed to elicit comprehensive and nuanced responses about their experience in healthcare access. The interviews focused on four key areas: access to healthcare services, the acceptability of these services within their communities, the affordability of obtaining care, and the overall availability of health services in their regions.

Further, this study utilized data from the 2020 SDHS, which is a comprehensive national survey designed to gather and analyze demographic data and healthcare utilization in Somalia. The primary goal of this data was to provide insights into the equity in healthcare access and understand pastoralists utilization of services. A comprehensive explanation of the SDHS methodology is available in previous publications [5, 16]. The SDHS used a multistage cluster sampling method to ensure the data collected was representative of the national population. To construct the sampling framework for the survey, 47 distinct strata were established. Utilizing high-resolution satellite imagery and on-the-ground insights, dwelling structures in urban and rural locations were digitalized. Enumeration Areas (EAs) were then delineated by spatially counting these structures with Geographic Information System (GIS) software. For the nomadic populations, the sampling frame was developed from an updated list that included 2,521 Temporary Nomadic Settlements (TNS), provided by nomadic network [5].

### Data analysis

In analyzing the Demographic Health Survey (DHS) data, we employed descriptive statistical methods, primarily focusing on frequency analysis using SPSS software. This approach allowed us to present the extent of healthcare utilization among pastoral communities. Our findings are systematically organized and presented in tables, which facilitate a clear and concise visualization of the data.

Regarding the qualitative data analysis, we draw inspiration from the Tanahashi framework for coverage [15], which has been duly adapted to the unique Somali context. The Tanahashi framework assesses the availability, accessibility, acceptability, and affordability of services [15].

Availability was explored to the extent to which the facilities have the necessary resources, such as personnel and health infrastructure, to meet the needs of the pastoralists.

Accessibility refers to geographic accessibility, which is determined by how easily communities can physically reach the health facility: many pastoralists live in areas with poor infrastructure, making travel to health care facilities challenging.

Affordability was determined by how the provider’s charges relate to the pastoralists’ ability to pay for services. For many in these marginalized communities, economic hardship may significantly impact their capacity to afford health care.

Finally, acceptability was explored to the extent to which the communities are comfortable with the characteristics of the services and how they fit to their needs. In Somalia, cultural sensitivity is vital as pastoralists may have specific cultural and social norms that impact their interaction with health care services. The Tanahashi framework was divided into supply and demand sides of the coverage [17, 18] as shown in Table 1.

**Table 1:** Mapping supply and demand determinants to the Tanasha’s framework

**Supply-side** factors within the health system, which pertain to the production and provision of healthcare services.	**Availability**: The provision of healthcare services is largely determined by the presence of essential resources, including healthcare workers, facilities, and medications. The extent to which these resources are available dictates the capacity to deliver healthcare effectively. **Accessibility**: This refers to the portion of the population that can utilize or reach healthcare services. For a service to be truly accessible, it must be geographically reachable—within a reasonable distance for those who require it. **Affordability** refers to the financial capacity of individuals and communities to access necessary services without experiencing economic hardship. It includes direct costs, like treatments and medications, and indirect costs, such as transportation.
**Demand side** determinants are factors operating at the individual, household, or community level that affect a person’s willingness to seek and utilize appropriate healthcare services.	**Acceptability** refers to the degree to which health services are perceived as suitable, respectful, and in alignment with the cultural and social norms of these communities.

Source; Henriksson et al. 2017 & McCollum, R. et al. 2019

We used thematic analysis, which is a robust qualitative research method used to identify, analyze, and report patterns within data [19–21]. The IDIs were first transcribed in Somali, translated in English and back-translated. The transcription and analysis were conducted by four researchers. To validate that each question was transcribed verbatim, the researchers reviewed the records several times. Afterwards, we began data coding, which involves subdividing the large amount of raw data and subsequently assigning them into categories. The categories were subsequently organized into specific themes [20], which were then mapped onto the four components of the Tanahashi framework (Table 5). The results are presented using direct quotes from participants. Each quote is labeled with a unique identifier, consisting of the participant number followed by a letter representing their region of residence. For instance, if a participant is from Galmudug, the identifier ends with “G”; if from Puntland, it ends with “P”; and if from Somaliland, it ends with “S”.

To ensure the trustworthiness and scientific integrity of our findings, we adopted a thorough approach that drew on the diverse experiences and perspectives obtained from multiple in-depth interviews [22]. Adhering to best practices for enhancing rigor in our study, we conducted member checking. This process involved in inviting three participants to review the accuracy and completeness of the transcriptions, ensuring that their viewpoints and intentions were correctly represented. Additionally, we employed investigator triangulation and reflexivity, with four researchers involved in the data analysis [22]. Their differing perspectives and insights contributed to a more enriched analysis and interpretation of the data. Before data collection commenced, we obtained ethical approval from the ethical board at Somali National University. Participants were fully informed about the study and were assured of their right to withdraw at any point without consequence.

## Results

Findings from the Demographic Health Survey (DHS) reveal that pastoral communities encounter more significant challenges across all access-barrier indicators compared to urban and rural communities in the country. Vast majority of pastoralists reported difficulties in securing funds for treatment (71.9%) and accessing health facilities due to distance (74%). These figures are higher compared to urban populations, with 58.5% and 51% respectively, and rural populations, with 66% and 62% respectively, as detailed in Table 2.

**Table 2:** Barrier indicators

Service Indicator	Response	Urban (%)	Rural (%)	Nomadic (%)
Problem in accessing health care	Getting permission to go for treatment	38.8	43.9	44.2
	Getting money for treatment	58.5	66	71.9
	Distance to health facility	51	62.2	73.8
	Not wanting to go alone	39.8	45.8	55.8
	At least one problem accessing health care	70.4	73.3	73.2

When examining reproductive indicators, pastoralist communities exhibit a high fertility rate, with an average of 7.3 children per woman, surpassing that of both urban and rural populations. Additionally, Table 3 highlights that the use of modern contraceptives among pastoralist women is exceptionally low, at just 0.1%. This figure is lower than the usage rates observed in urban areas (2.1%) and rural regions (0.5%).

**Table 3:** Reproductive indicators

Service Indicator	Response	Urban	Rural	Nomadic
Total Fertility Rate	TFR per women (15–49)	6.4	7.1	7.3
Birth spacing	Any modern method	2.1	0.5	0.1

Similarly, Table 4 reveals significant disparities in healthcare access between pastoralists and urban populations. Among pastoralists, 90% do not utilize antenatal care services, in stark contrast to 49.2% in urban group. When it comes to childbirth, only 8.3% of pastoralist women have the assistance of a skilled birth attendant, compared to 51% of women in urban areas. Additionally, 4.3% of pastoralist women give birth in healthcare facilities, and 84.2% of their children do not receive essential vaccinations like BCG, DPT, Measles, and Polio. In comparison, 33.7% of urban women deliver in health facilities, and 39.6% of their children miss out on these vaccinations.

**Table 4:** Maternal and child health service indicators

Service Indicator	Response	Urban (%)	Rural (%)	Nomadic (%)
Antenatal Care	None	49.2	63.9	90
	1	6.8	6.1	4.6
	2 to 3	29.8	19.5	4.4
	4+	13.7	10.2	0.8
	Don’t know / missing	0.5	0.3	0.2
Assisted Delivery	Skilled Birth Attendance	51	36.9	8.3
Place of Delivery	Delivered in health facility	33.7	24.7	4.3
Postnatal check	Less than 4 h	16.8	9	1.9
	4–23 h	1.2	1.1	0
	1–2 days	0.6	0.8	0.6
	3–6 days	0	0.1	0
	Don’t know	0.5	0.4	0.1
	No postnatal check	80.5	88.5	97.3
PNC with 2 days		18.7	11	2.6
Vaccination	All basic vaccination (BCG, DPT, Measles, Polio)	18.7	14.5	0.6
	No vaccination – Zero dose children	39.6	52.7	84.2

### Qualitative study findings

As highlighted in Table 5, the study identified nine themes, which were organized under the four categories of the Tanahashi framework, which comprises four critical dimensions: availability, accessibility, acceptability, and affordability of healthcare services.

**Table 5:** Themes that emerged from the interviews

Services	Tanahashi framework	Themes
Supply	Availability	Struggling without services
		The devastating impact of service shortfalls
	Accessibility	Transportation in emergency situations
		Urgency and distance
		Mobile lifestyle
	Affordability	The financial strain of transport
		Priced out of healthcare
		The sacrifice of livestock for health
Demand	Acceptability	Cultural connection

### Supply side: Availability

#### Struggling without services

The availability of healthcare services in pastoralist communities, as reflected by participants, highlights a profound insufficiency and inequity in availability of services. The absence of healthcare facilities that are within reach is a major challenge, forcing pastoralists to travel long distances to the nearest center. This scenario exemplifies the critical shortage of healthcare infrastructure, health providers and essential treatment in pastoralist areas, which poses significant risks to the health and well-being of these communities. The observed disparity in healthcare availability suggests that resource allocation heavily favors urban centers, leaving pastoralist communities marginalized, which may compel pastoralists to rely on traditional birth attendants for essential healthcare services.

*Our village completely lacks healthcare services. We have a traditional birth attendant who was invaluable to us*,* akin to gold. Unfortunately*,* she is no longer active due to her blindness. (P2S)*

*This area is devoid of hospitals and lacks sufficient healthcare services to meet the community’s needs. (P7P)*

*There are no health services specifically aimed at pastoralists*,* whether provided by the government*,* NGOs*,* or any other entities. For any health needs*,* we are compelled to travel to urban centers. (P5G)*

#### The devastating impact of Service Shortfalls

The consequences of lack of availability of health services for pastoralist communities in Somalia, as illustrated by participants are both tragic and severe. Vast majority of participants mentioned family members who died for lack of healthcare intervention. The story of a woman who suffered from heavy bleeding and died on her way to far away healthcare facility, and another that passed away because of lack of transportation underscores the critical impact of delayed medical intervention due to the absence of nearby healthcare facilities. These cases highlight how life-threatening conditions, which require immediate medical attention, often result in fatalities because essential services are not available within a critical timeframe. Similarly, the account of a family member who died during labor due to the unavailability of healthcare staff in pastoralist areas further illustrates the dire outcomes of inequitable health system. The geographical isolation of these communities compounds these issues, as the lengthy distances to medical facilities not only delay needed intervention but also significantly elevate the risk of severe complications or death.

*Recently a woman… suffered heavy bleeding… taken to “Toon”*,* but she passed away on the way. (P2S)*

*Because we are very far from health facilities*,* a person who becomes seriously ill cannot reach medical care in time. The long distance often delays treatment*,* which increases the risk of complications or even death. (P8G)*

### Supply side: Accessibility

#### Transportation and emergency situations

The transportation difficulties consistently emerge as an immediate barrier, transforming even routine medical needs into crises. In emergencies, the urgency of reaching healthcare facilities is thwarted by the absence of vehicles, forcing desperate measures like traveling on foot. Such conditions are especially perilous, as delays exacerbate health issues, turning potentially treatable conditions into critical situations. The emotional toll is further highlighted by the recurring stress and anxiety experienced by community members, like one parent facing repeated childbirth emergencies under these strained circumstances. This finding underscores the urgent necessity for targeted interventions to bridge these critical gaps in healthcare accessibility and ensure timely medical attention for pastoralist populations.

*If we don’t have a vehicle to help us transport the patient*,* we must urgently call someone for help. I once had to rush my daughter to “Dhimriyaale” and then called a female doctor from Hargeisa*,* which is far away. I have been through this situation three times*,* each time facing the same challenges when my daughter gave birth.* (P2S)

*We have to either hire a vehicle for transport or rely on walking if no other option is available. Unfortunately*,* we lack a dedicated transportation service. Just last night*,* a member of my household fell seriously ill. We faced a 12-hour ordeal trying to care for the patient at home because we couldn’t secure a vehicle during the night. It was only by morning that I finally managed to arrange transportation to take the patient to the nearest health center. (P7P)*

#### Distance to Health Facilities

The distances pastoral communities must travel to reach healthcare facilities are alarmingly vast, revealing a critical challenge in accessing timely medical care. With the nearest centers like “Toon” being a full day’s walk away, reaching medical help is a formidable task, especially for those in critical condition. In many cases, like the journey to “Habaaswein” or a healthcare facility 120–130 km away, the logistics of securing a vehicle can further delay treatment, risking patients’ health and safety. Even a relatively closer facility, situated 60 km away, presents a daunting challenge, particularly during emergencies when time is of the essence. These distances not only hinder urgent healthcare access but also discourage preventive care and routine check-ups, leaving many health issues untreated until they escalate.

*The nearest place is “Toon”*,* but it’s far from here. Walking to the nearest place can take a whole day*,* which is impossible for critical patients. About a full day’s walk to reach “Toon” or “Habaaswein*”(*P1S)*

*The nearest healthcare facility is 120–130 kilometers away. When we request a vehicle*,* it sometimes takes a whole day to arrive*,* and in emergencies*,* the patient may deteriorate before reaching care. (P5G)*

*The nearest proper healthcare facility is located about 60 kilometers away. This long distance poses a huge challenge for patients*,* especially in emergencies. (P7P)*

#### Mobile lifestyle

For some pastoralists their lives are marked by constant movement. Despite their inherent mobility, pastoralist communities are living in remote areas, further complicating healthcare access. The combination of mobility and living in remote areas necessitates innovative healthcare solutions that can cater to their unique needs and ensuring that even the most remote pastoralists receive the healthcare they require.

*As pastoralists who move from place to place*,* there is no health facility we can rely on for healthcare. Yet such services are crucial for our survival given our lifestyle and constant mobility (P6P)*

*Health facilities are located far from this area*,* and even the nearby villages lack healthcare facilities. No matter where we move*,* we must seek healthcare services in towns that are far from here (P11G)*

### Supply side: Affordability

#### The financial strain of transport

The high transportation costs were reported to be particularly burdensome for pastoralist communities, who may already face economic constraints due to their reliance on livestock and the impacts of environmental factors such as drought. The need to rent vehicles for emergency or planned medical trips places financial strain on these communities. The costs, ranging from 200 to 300 dollars, are considerable for families whose livelihoods depend on livestock, which can be unpredictable in terms of income.

*The transportation cost for such a journey is extremely high… when we have to go all the way to Hargeisa*,* the amount we are charged is very large. (P2S)*

*We do not have transport for a sick person. The patient may remain without care for a long time…; We rent vehicles here ourselves. It costs us at least 200 to 300 dollars. It is a hired vehicle. (P12P)*

#### Priced out of healthcare

The participants highlighted the substantial barriers to healthcare faced by pastoralist communities, primarily due to high costs. The absence of free healthcare options exacerbates this issue, forcing individuals to make difficult decisions between essential needs and medical care. This financial strain is evident across various healthcare needs, from emergencies to specialized services, and even postpartum care, where high costs prevent malnourished mothers from receiving necessary nutritional support. The overall inaccessibility of affordable healthcare leads to untreated illnesses and deteriorating health within the community, underscoring the urgent need for systemic reforms to create equitable and sustainable healthcare access.

*Healthcare is like a business*,* if you have money*,* you will be served. If you don’t*,* then it is not possible to receive treatment. There is no support system for poor pastoralists. (P5G)*

*We do not have enough money*,* and there are no free hospitals. Sometimes we can afford healthcare*,* but other times it is beyond our means. When healthcare is not accessible and there is no money*,* a sick person simply remains at home in the community without receiving treatment. Healthcare costs are high*,* especially when specialized services are required.”(P8G)*

*The cost involved is quite high. Even when I am in the postpartum period*,* I have to leave without receiving nutritional support because of the high expenses. (P2S)*

#### The sacrifice of livestock for health

The participants underscored the reliance of pastoralist communities on livestock as a critical resource for managing health issues when they arise. In the absence of adequate support systems and faced with prohibitive healthcare costs, pastoralists often resort to selling livestock to cover medical expenses. This practice highlights the precarious balance they must maintain between sustaining their livelihoods and addressing urgent health needs. However, the economic constraints are significant, and even the sale of livestock may not always generate sufficient funds to afford healthcare, particularly for treatments that are costly or specialized. This situation illustrates the broader economic vulnerabilities faced by pastoralists and the need for more robust support systems and affordable healthcare options to reduce their reliance on livestock sales and improve health outcomes within these communities.

*As pastoralists*,* we handle health issues independently*,* addressing problems as they arise. When health needs arise*,* we try to manage them using available resources*,* such as livestock or other support accessible at the time. Healthcare costs are frequently beyond our financial reach*,* making it challenging or sometimes impossible to afford essential treatment. (P6P)*

### Demand side: Acceptability

#### Culturally Connected Healthcare Providers

While accessing healthcare presents significant challenges for pastoralist communities, the acceptability of services once they are reached is generally positive. The fact that healthcare providers are often part of the community themselves fosters a cultural respect and understanding that enhances the acceptability of their services. Participants reported that healthcare providers are part of their community, leading to a respectful understanding of and familiarity with their culture. The culturally sensitive providers can deliver care in a culturally tailored manner, which is vital for ensuring that pastoralists feel comfortable and respected during medical interactions. However, the lack of healthcare providers in pastoralist areas means that these positive relationships and culturally respectful services remain elusive for most.

*The healthcare providers offer tailored services*,* but we must travel a significant distance to access them and receive the care. (P13P)*

*There is no patient-provider relationship here because we have no provider in the area.* (*P1L)*

## Discussion

Our analysis of demographic health surveys reveals notable disparities in health service utilization, with pastoralist communities exhibiting the lowest levels across all domains. Over 70% of pastoralists report facing substantial challenges in securing funds for medical treatment and accessing healthcare facilities due to long distance to facilities. This financial and geographic inaccessibility hinders their ability to obtain necessary care. As a result, vaccination rates among children are low, and women’s access to essential services, such as antenatal care, is severely limited, with 90% of women not receiving these vital interventions. These barriers underscore the urgent need for targeted interventions to enhance healthcare access and improve health outcomes for pastoralist populations. The 2020 Somalia Demographic Health Survey report provides a comprehensive detailing of the disparities in health service utilization across communities, highlighting the profound inequities faced by pastoralist populations [6]. To gain an in-depth understanding of healthcare access from the perspective of pastoralists, we employed the Tanahashi framework for coverage, which utilizes a systems thinking approach. This framework allowed us to thoroughly explore the various dimensions of healthcare accessibility among pastoralist communities. By doing so, we were able to capture extensive explanations in their own words, revealing the complexities and challenges involved in accessing health services. The findings reveal that pastoralist communities, which make up approximately 30–35% of the population, are largely deprived of essential healthcare services across the three states that we studied. Studies shows that nomadic pastoralist communities continue to experience lack of access to healthcare, and subsequent elevated levels of illness and death due to various conditions that could be prevented [7, 23–25]. We explored the availability of services which refers to both the physical presence of health facilities, workforce, and supplies in pastoralist areas and the capacity of those facilities to provide necessary care [26]. The study found that health services are not available for pastoralist in Somalia with this finding being constant in the three regions involved in this study. In line with our findings, an earlier study among pastoralists in Somalia identified various obstacles in health service availability for pastoralist women and children [27]. The findings emphasize critical deficiencies, such as a lack of essential supplies, inadequate staff training, and the unavailability of vital resources in maternal and child health centers [7]. The lack of health services in pastoralist areas has led to significant consequences, including delays in receiving care due to travel difficulties and increased personal health expenses for families already facing poverty. These challenges have ultimately contributed to shorter life expectancies within pastoralist communities, as individuals struggle to access timely and affordable medical care [28].

Regarding accessibility of the services, we found that long distance to health facilities, lack of transportation and mobile lifestyle of the pastoral communities affect their accessibility to tailored healthcare. This challenge is exacerbated by limited transportation options, poor infrastructure, and economic limitations, all of which impede timely and adequate care. The issue of limited healthcare access among Somali pastoralists has been well-documented in both earlier literature [2, 9, 23, 29] and more recent studies [7]. Consistent with our findings, previous research on Somali pastoralists revealed that the considerable distance to healthcare facilities often compelled these patients to sell substantial numbers of livestock to cover the high costs of seeking healthcare services. Such severe financial pressures may discourage individuals from seeking professional healthcare, prompting a reliance on traditional remedies instead [12]. A prior study challenges the common belief that Somali pastoralists migrate randomly using unpredictable routes, instead demonstrating that the migration patterns of Somali pastoralists are predictable as they travel between two clearly defined grazing areas [12]. An important takeaway from that study is that pastoralists can effectively integrate with conventional healthcare service delivery systems with potential to benefit from fixed healthcare services when these are strategically located along their migration routes [12]. Despite Somalia’s ambition to achieve universal health care, the inadequate access to health services for pastoralist communities presents a substantial obstacle to realizing this vision of comprehensive health coverage. Further, evidence shows that Somalia faces significant challenges in improving its critical health indicators, including elevated maternal and child mortality rates, a high prevalence of tuberculosis, and the widespread incidence of neglected tropical diseases [6]. Given our findings, without addressing the disparities faced by pastoralist communities, progress towards improved health outcomes may remain stalled. It is therefore imperative that Somalia’s health system undergoes substantial reform, adopting an equity perspective to ensure that all citizens, regardless of their geographic location or lifestyle, have access to necessary healthcare services.

According to our findings, the affordability of health services for pastoralists remains a significant issue, primarily due to factors such as pervasive poverty, high healthcare costs, insufficient financial resources, and income fluctuations caused by environmental challenges like droughts [8]. In pastoralist communities within Somalia, the financial barriers to healthcare are so severe that households often resort to selling their livestock to cover medical costs [12]. This practice compromises the very foundation of their livelihoods, as livestock constitutes a critical asset and economic lifeline for these communities. The necessity to liquidate such essential resources underscores the acute affordability challenges they face, where the high out-of-pocket expenses for healthcare services and medications are insurmountable without sacrificing their primary means of sustenance. As a result, the choice between accessing healthcare and maintaining their basic livelihood presents a profound dilemma, highlighting the urgent need for interventions to improve healthcare affordability in these communities.

Acceptability of healthcare among pastoralists refers to the degree to which health services are perceived as suitable, respectful, and in alignment with the cultural and social norms of these communities. It encompasses the willingness of individuals to seek healthcare services based on how well these services fit with their values, beliefs, and lifestyle. Most participants in our study believed that the services they receive are in alignment with their cultural and social norms. Despite the critical importance of ensuring cultural sensitivity among healthcare providers, which can enhance the acceptability of healthcare services, this alone does not address the significant barriers facing pastoralist communities in Somalia. When fundamental components of healthcare coverage, such as availability, accessibility, and affordability are missing, the value of cultural sensitivity and acceptability may become negligible. In such scenarios, even culturally attuned healthcare services may fall short of meeting the needs of pastoralist populations if they are physically inaccessible or financially out of reach. Therefore, while cultural sensitivity in healthcare provision is important, addressing the broader systemic issues of service availability, accessibility and affordability is crucial to ensure meaningful healthcare coverage for pastoralist communities.

Finally, the findings showed that the health inequity in Somalia is a pervasive issue, particularly affecting pastoralist communities who remain marginalized and largely excluded from adequate healthcare services [7]. This disparity is not merely a matter of healthcare access but represents a fundamental barrier to achieving broader national and international objectives [30]. The exclusion of significant pastoralists from healthcare, undermines Somalia’s vision of universal healthcare; an ambition that aims to ensure all individuals have access to necessary health services without financial hardship [30, 31]. Moreover, the observed inequity presents substantial obstacles to achieving several Sustainable Development Goals (SDGs), such as those related to good health and well-being for all, reducing inequalities, and eradicating poverty. Thus, reforming the healthcare system to address the needs of pastoralist communities is not only crucial for the realization of universal healthcare but also essential for meeting the broader spectrum of sustainable development objectives. Such reform requires a multi-faceted approach, including enhancing service availability in pastoralist areas and ensuring financial protection to make healthcare affordable for pastoralists. Prioritizing these reforms is imperative for aligning Somalia’s health system with its national and international commitments and for fostering an inclusive society where no community is left behind.

The study has both strengths and limitations. The study’s strength lies in its use of a mixed-methods approach, combining representative register data with qualitative exploration to highlight the inequities Somali pastoralists face in accessing healthcare. The representative register data provided quantitative analysis, offering statistical evidence of healthcare access disparities across different settlements. This quantitative foundation was complemented by qualitative exploration, which offered deeper insights into systemic, social, and logistical barriers that affect healthcare access, capturing the lived experiences and perceptions of the pastoralist communities. A notable strength of our qualitative part of the study is the open and unstructured nature of interviews, allowing participants to express themselves freely without being constrained by predetermined questions. Many shared perspectives and opinions were consistently echoed by various participants. The weakness include that we interviewed pastoralists without including healthcare providers and policymakers, potentially overlooking critical information regarding healthcare access.

## Conclusion

Given the significant disparities in healthcare access in Somalia, it is imperative to undertake comprehensive health system reforms that inclusively integrate pastoralist populations into health planning strategies. This integration is crucial for addressing the unique needs of these communities, who are often marginalized in conventional healthcare frameworks. We recommend a national initiative to meticulously map the migration patterns of pastoralists. Such an undertaking would enable the design of healthcare services that are not only available and accessible but also culturally acceptable and affordable to pastoral communities. By aligning healthcare service delivery with the migration routes of pastoralists, we can enhance their access to healthcare and promote equitable health outcomes. A future study that explores the perspectives of healthcare providers and policymakers is required to gain a more comprehensive understanding of the healthcare challenges faced by pastoralist communities.

## Supplementary Information

Below is the link to the electronic supplementary material.

Supplementary Material 1

## Data Availability

The data that support the findings of this study are available from Somali Institute for Health Research (SIHR), but restrictions apply to the availability of these data, which were used under licence for the current study and so are not publicly available. The data are, however, available from the authors upon reasonable request and with the permission of Somali Institute for Health Research (SIHR).
