# Supplementary material for: Unveiling healthcare disparities in Somalia: the hidden struggles of nomadic communities
Source: Int J Equity Health. 2026 Apr 21;25:141. doi: 10.1186/s12939-026-02846-7 (PMC13235041; doi:10.1186/s12939-026-02846-7)
Supplement: Supplementary file 1 — Supplementary Material 1 [file 12939_2026_2846_MOESM1_ESM.pdf]

Question guide for pastoralists

Access to Healthcare

1. Personal Experience
   - Can you describe your experience in accessing healthcare services in your area?
   - Have you ever faced any barriers when trying to access healthcare? If so, what were they?
   - What challenges do you face in accessing healthcare services? For instance, distance, cost, cultural barriers, etc.?

2. Availability of Services
   - What healthcare facilities are available within your locality? Did they provide enough services?

   - Are specialized medical services available in your area? How do you feel about the availability of specialized medical services in your area?

3. Geographical, conflict and climate change related Barriers
   - How far is the nearest healthcare facility from your home?
   - Can you talk about the challenges posed to you by the distance to healthcare facilities?
   - Can you describe an experience where your efforts to seek healthcare were disrupted by a conflict situation
   - Can you describe a time when you had to move to a location far from healthcare facilities because of climate change-related droughts, which made it difficult for you to access medical care?

4. Transportation
   - How do you reach your nearest healthcare facility? Do you have reliable transportation to get to healthcare appointments?
   - How does the availability (or lack) of public transportation affect your ability to access healthcare?

Acceptance of Healthcare

1. Trust and Perception
   - Can you describe your general perception of the healthcare services you receive?
   - How is your trust towards the healthcare providers in your community?

2. Cultural Competency
   - How do healthcare providers address your cultural or personal preferences during treatment?

3. Patient-Provider Relationship
   - How would you describe your relationship with your primary healthcare provider?
   - How do you feel comfortable discussing your health concerns with your healthcare provider?
4. Inclusivity
   - How well do healthcare providers in your area address the needs of pastoralist populations and how are services aligned with your needs?

Affordability of Healthcare

1. Costs
- How do you pay your healthcare expenses?
- How manageable are your healthcare expenses given your current financial situation?

2. Financial Barriers
- Can you tell me a time when you delayed or foregone medical treatment because of cost?
- How do out-of-pocket expenses affect your ability to seek care?

4. Comparative Costs
- How do you feel about the cost of healthcare services compared to other necessary expenses in your life?
